# Association studies between chromosomal regions 1q21.3, 5q21.3, 14q21.2 and 17q21.31 and numbers of children in Poland

**DOI:** 10.1038/s41598-022-21638-x

**Published:** 2022-11-07

**Authors:** Jeremy S. C. Clark, Thierry van de Wetering, Błażej Marciniak, Elżbieta Żądzińska, Andrzej Ciechanowicz, Mariusz Kaczmarczyk, Agnieszka Boroń, Kamila Rydzewska, Konrad Posiadło, Dominik Strapagiel

**Affiliations:** 1 Department of Clinical and Molecular Biochemistry, Pomeranian Medical University, Szczecin, Poland; 2 Biobank Lab, Department of Molecular Biophysics, Faculty of Biology and Environmental Protection, University of Lodz, 90-237 Lodz, Poland; 3 Department of Anthropology, Faculty of Biology and Environmental Protection, University of Lodz, 90-237 Lodz, Poland; 4 Biological Anthropology and Comparative Anatomy Research Unit, School of Medicine, University of Adelaide, Adelaide, SA 5005 Australia

**Keywords:** Biomarkers, Genetic markers, Biomarkers

## Abstract

Number of children is an important human trait and studies have indicated associations with single-nucleotide polymorphisms (SNPs). Aim: to give further evidence for four associations using a large sample of Polish subjects. Data from the POPULOUS genetic database was provided from anonymous, healthy, unrelated, Polish volunteers of both sexes (N = 5760). SNPs (n = 173) studied: (a) 69 from the chromosome 17 H1/H2 inversion; (b) six from 1q21.3, 5q21.3 and 14q21.2; and (c) 98 random negative controls. Zero-inflated negative-binomial regression (z.i.) was performed (0–3 numbers of children per individual (NCI) set as non-events; adjustors: year of birth, sex). Significance level *p* = 0.05 with Bonferroni correction. Statistically-significant differences (with data from both sexes combined) were obtained from highly-linked inversion SNPs: representative rs12373123 gave means: homozygotes TT: 2.31 NCI (n = 1418); heterozygotes CT: 2.35 NCI (n = 554); homozygotes CC: 2.44 NCI (n = 43) (genotype *p* = 0.01; TTvs.CC *p* = 0.004; CTvs.CC *p* = 0.009). (Male data alone gave similar results.) Recessive modeling indicated that H2-homozygotes had 0.118 more children than H1-homozygotes + heterozygotes (z.i.-count estimates ± standard errors: CT, − 0.508 ± 0.194; TT, − 0.557 ± 0.191). The non-over-dispersed count model detected no interactions: of importance there was no significant interaction with age. No positive results were obtained from negative-control SNPs or (b). Conclusions: association between the H1/H2 inversion and numbers of children (previously reported in Iceland) has been confirmed, albeit using a different statistical model. One limitation is the small amount of data, despite initially ~ 6000 subjects. Causal studies require further investigation.

## Introduction

Numbers of children (as well as the timing of births, changes in sex ratio at birth etc.) are important human traits, particularly relevant to public health (e.g., see Żądzińska et al.^1^; Jasieńska et al.^2^).

The present study analyzes several associations between single nucleotide polymorphisms (SNPs) and numbers of children which have been detected from various populations in two large studies: Stefansson et al.^3^ and Barban et al.^4^. The functional significance of these SNPs is mostly unknown but this is not a bar to further statistical analysis as functional studies can follow later.

It is of great value, regardless, to produce confirmatory replications for all associations involving human subjects, not only because of the high prevalence of false positives due to unknown sources of bias (see^5^), but also because of the possibility of differences between populations^3^. In general a reproduction study (a replication performed by another Institute) is only useful if the result is positive, because a negative result can always be assumed to have arisen because of differences in methodology, population, some other systematic difference or, especially if the reproduction has less subjects, lack of power or random error.

In order to replicate genotype–phenotype association studies exactly there are several requirements: (1) the phenotypic parameter needs to be defined and data collected in the same way; (2) the genotypic change needs to be thoroughly understood; (3) the numbers of subjects should be at least as large; and (4) the exact regression model should be replicated.

In the presented study all four requirements present hurdles, as described below and in the Discussion, which means that the final results cannot be said to be exact reproductions of the initial results from the two original publications but, even so, can contribute to an evidential basis confirming association, if positive.

### Phenotype

At first sight, genetics might not seem likely to play a major role in numbers of children, but it must be remembered that the extent to which human behavior is affected by genetics has not been fully clarified. To resolve such questions huge amounts of data are required, even for quite large effect sizes, in order to counter interacting effects and bias, due e.g. to socio-economic changes etc.. Even so, genome-wide association studies have identified at least 12 loci influencing human reproductive behavior^4^. It should also be noted that it is difficult to disentangle effects due to variation in fertility from variation in human reproductive behavior, and any associations found would need further studies in order to do this.

Year of birth (with identical analysis to age) is likely to have considerable effect on numbers of children, not only because of changes in fertility with age, but also because socio-economic changes have brought about considerable increase in reluctance to have more than a few children, whereas previously large families were common. There are numerous demographic effects which have contributed to this, including the availability of contraception, which mean that human reproductive behavior has varied considerably over the time period studied. In addition there are multivarious factors, such as obesity^6^, excessive exercise, smoking, alcohol consumption and sexually transmitted diseases which all negatively affect the fecundity of females and males^7–10^, and which also varied over the time period.

It was decided that, even if positive results were obtained for association between number of children per individual (NCI) and a genotype, these would be regarded as negated by the presence of significant interaction with age because of the demonstrably high correlation between numbers of children and year of birth.

### Genotype

Most SNPs are well defined, but here SNPs have been studied which either (1) lie on a 900 kb inversion on chromosome 17 or (2) are linked surrogates to those studied by Barban et al.^4^.

1. The H1_H2 inversion.

The H1 and H2 versions of the 900 kb inversion at chromosome 17q21.31 were described by Stefansson et al.^3^ and in the present study were identified using SNP alignments. The inversion has boundaries that are not necessarily easy to define using SNPs: Stefansson et al.^3^, using microsatellites and bacterial artificial chromosome-based contiguous (BAC) sequences, showed that the two main haplotypes, H1 and H2, do not align exactly on respective chromosomes and, have associated duplicated region(s). A duplication will interfere with SNP analyses, meaning that it is safer to analyze SNPs covering a slightly smaller region than the whole inversion including flanking regions.

Stefansson et al.^3^ showed that the H1 and H2 lineages diverged around 3 million years ago, with H2 probably having an African origin. H2 was found to have extreme homogeneity among subjects with H1 relatively diverse. The strongest linkage disequilibrium was found in a Utah sample (with genetic relationships with Slavic Europeans) indicating very little, if any, recombination in the European population. No recombination, despite sufficient prevalence of H2 homozygotes, suggests recent positive selection for H2 and, probably, rapid expansion in Europe, including in the Icelandic population studied by Stefansson et al.^3^. This provides the original hypothesis that the inversion might be associated with numbers of children.

Steinberg et al.^11^ used next-generation sequencing, BAC-based assemblies and fluorescence in-situ hybridization (FISH) to explore the architecture of the 17q21.31 region, especially in African populations, and found eight structural configurations based on duplication content and organization. They identified 887 SNPs which could distinguish the two variants (Stefansson et al.^3^ identified 36 SNPs) and concluded that an ancestral H2' variant (' = without a duplication) was replaced almost entirely by an H1' variant. Subsequently, duplication events occurred separately in H1 (205 bp; H1D) and H2 (155 bp; H2D) and these are now found at high frequencies within European but not Asian or African populations. In the present study a range of SNPs were used to identify the extent of non-recombinant regions and therefore the two main inversion variants in a Slavic population.

2. The Barban SNPs.

In a genome-wide association study (GWAS) by Barban et al.^4^, three SNPs were found to show significant associations with numbers of children: rs10908474, rs13161115 and rs2415984 (on chromosoms 1, 5 and 14, respectively); referred to here as the "Barban SNPs". The *p*-values for these associations were: for female and male data together: rs10908474, *p* = 2.08 × 10^−8^; rs2415984, *p* = 2.34 × 10^−8^; from male data only: rs13161115, *p* = 1.37 × 10^−8^. As these were below the significance level commonly used for GWASs (*p* = 5 × 10^−8^), Barban et al.^4^ searched for the nearest genes which might be involved in male fertility using many complex methods, but no firm conclusions were drawn. The three Barban SNPs could not be found in the POPULOUS database and so in the present study six surrogate SNPs were selected using logarithm of odds linkage scores (see Methods).

### Regression and sample size

Regression model presentation has recently improved, for example with the availability of the R statistical platform^12^, where advanced regression models are freely available.

Stefansson et al. (2005)^3^ had analyzed 16 959 females and 12 178 males (5463 families with 29 137 individuals) born between 1925 and 1965 and examined the relationship between H2 genotypes and numbers of children in the Icelandic population. In Stefansson et al.'s Supplemental Fig. 7b^3^ an expected distribution for numbers of children is found with a peak at two children (for reports from males). However, Supplemental Fig. 7a from the same source shows a very unusual distribution with probably a peak at four children with reports from females (the fifth value shows ≥ 5 children; the number with exactly five children, and subsequent values, are almost certainly less). Assuming a dominant effect (with data from homozygotes and heterozygotes summed) with weighted, multiple (presumably linear) regression adjusted for year of birth, they showed that for both sexes combined H2 homozygotes + heterozygotes had more children than H1 homozygotes. (Their study group contained related individuals and therefore standard errors and *p*-values were calculated using a permutation technique).

Barban et al.^4^ used association models with various adjustors: year of birth (and square or cube of this to control for possible non-linear birth cohort effects) and principal components to reduce confounding by population stratification. This was a huge study, with 343 072 subjects and 62 cohorts from 17 countries (from Australia, Europe and the USA), which was replicated internally with quality control conducted in two independent centers. Results were filtered using the QC protocol (QCGWAS16 and EasyQC17 filters) of the GIANT consortium^13^ and subsequently meta-analyzed (using a sample-size-weighted-fixed-method) for the 2.4 million SNPs that had passed the filters. SNPs with the lowest association *p*-values, indicated by multiple linear regression under an additive model, showed independent loci significantly associated with "number of children ever born" (NEB; which includes fetal deaths and still-births).

It can be surmised that the tendency to have two or three children is, nowadays, so enforced by social norms that many couples try very hard to achieve this. However, some people quite possibly escape these pressures and after a certain number of children might not care so much about exactly how many children they have. It is this escape which becomes of interest if it can be shown to be genetically linked. By using zero-inflation models it is possible to set a non-event as being zero, one or more children. In the present article it was decided to set a non-event as being 0 to 3 children, three being the upper integer bound to the mean number of children. This is an important assumption intended to highlight possible differences between groups in terms of the numbers of individuals with more children than average.

The hypothesis was that one or several of the SNPs previously suggested to have association with numbers of children (and/or surrogates close to these SNPs) could be shown to give statistically significant associations with numbers of children (above three) per individual in the Polish population.

The aim of study was therefore to implement a zero-inflated negative binomial regression model to study association between frequency counts of SNPs, identical or closely-linked to SNPs previously associated with NEB by Stefansson et al.﻿^3^ or Barban et al.^4^, with the numbers of children per individual, adjusted for year of birth and sex (and interactions). SNPs on chromosomes 1, 5, 14 and 17 were analyzed, as well as ~ 100 random negative control SNPs, using data from a large exomic SNP database collected from the entire geographical region of the Polish population.

## Materials and methods

### The POPULOUS database

Access to the POPULOUS database was granted for this study following a license agreement (PUM UL 001) between the University of Łódź (UL) and the Pomeranian Medical University (PUM), Szczecin, Poland. This database was the outcome of the TESTOPLEK project (funded by the European Union); approved by the UL Ethical Review Board, and all procedures were in accordance with the current Declaration of Helsinki (2013). Genetic data was made available from anonymous Polish unrelated volunteers, who had declared themselves as healthy and signed written informed consent.

The question used to provide the phenotype "number of children per individual" (NCI) was simply "how many children have you had ?" [in Polish]. NCI is related to "number of children ever born" (NEB) but excludes fetal deaths and still-births.

Procedures used to collect samples, DNA isolation and genetic analysis can be found within the POPULOUS project description^14^. SNPs were selected which passed project quality-control criteria and had minor allele frequency (MAF) > 1% (to give "Populous data"). Genotype data were originally written onto Genome Studio software (Illumina, San diego, CA, USA; https://www.illumina.com) and were exported using a PLINK INPUT report plug-in (plink-input-report-plugin-v2-1–4; Illumina) into .MAP and .PED files which could be easily imported into R^12^ and/or PLINK (version 1.9 64-bit; www.cog-genomics.org/plink/2.0;^15^). Illumina SNP names, and NCBI dbSNP rs numbers if available, were downloaded from the manufacturer's website (infinium-coreexome-24 -v1-1-support-files; Illumina). Phenotype data were: sex (Female (1), Male (2)), "Age", district location ("county"), and the number of children per individual ("NCI"). Age (which analyzes identically with year of birth) was calculated by subtracting the year of birth from 2012; phenotype data was for the status of the patient in the year 2012.

### AgeRange selection

Age ranges included were ≥ 45 for data from women (n = 1219) and ≥ 55 for men (n = 728). These ranges follow Barban et al.^4^ and considerably reduce the chance that an individual might have more children after the survey.

### Inversion SNP lists

Initial inversion SNP lists were established based on the position of the two microsatellite markers D17S810 and D17S791 i.e. Region 1 (between 17:43488002 and 17:44857562; all chromosomal locations use GRCh37.p13) which included flanking regions on either side and 177 SNPs identified in the database. The inversion can also be enclosed by the closer microsatellite markers DG17S332 and DG17S161 and Region 2 (between 17:43793582 and 17:44776837) was established including very small flanking regions: 155 SNPs were identified in this region. SNP exclusion criteria: small insertion/deletions; one allele type (SNP base) for all 5642 subjects; < 75% subjects genotyped. Inclusion criteria (before alignment) included all SNPs not excluded: 118 SNPs for Region 1 and 99 SNPs for Region 2. (Following alignment, see below, SNPs at the edges, possibly within the flanking regions and suspected to lie outside the inversion region and which did not align, were also excluded.)

### Alignment of inversion SNPs

Diploid SNP bases, e.g. homozygote [A];[A], heterozygote [C];[T] and homozygote [C];[C] etc. are abbreviated in this article as AA, CT and CC etc. For each subject an artificial sequence was generated with all selected diploid SNP bases concatenated in the same order as on an H1 chromosome and these were uploaded into an online multiple alignment tool (MAFFT version 7; https://mafft.cbrc.jp/alignment/software/tips.html;^16^; scoring matrix 1PAM/κ = 2). The .aln file results were uploaded to jalview (version 2, https://www.jalview.org) for visualization: with red for A (adenine), green for C (cytosine), blue for G (guanine) and yellow for T (thymine). Patterns were identified (without colors, this would be almost impossible): most importantly to see if groups of subjects could be separated with many SNPs as either homozygous or heterozygous, and where these relationships broke down e.g. in border regions (perhaps associated with a duplication).

An initial SNP group was selected (from 99 SNPs) with a coverage of 100% i.e. with no missing data. Remaining SNPs were placed into the alignment regardless of the presence of missing data, without changing the order of the subjects. Those SNPs for which all subjects (ignoring missing data) were homozygous within one group of subjects, heterozygous in the second group, and counter-homozygous in the third group, were considered most interesting as these represented non-recombinant groups. The H1-H1 genotype was assumed to be represented by the homozygous group with the largest number of subjects (see Discussion). Small numbers of subjects or SNPs which did not fit into the major categories were excluded.

### Selection of Barban SNP surrogates

For each Barban SNP, two surrogate SNPs were selected in flanking regions up to 50 000 bp on either side of the investigated SNP and logarithm of odds (LOD) scores identified in Haploview (https://www.broadinstitute.org/haploview;^17^). The population chosen (EUR') for analysis was most similar to that in Barban et al.^4^ and included data from England (GBR), Spain (IBS) and Italy (TSI). The two surrogate SNPs which had genotype frequencies consistent with Hardy Weinberg equilibrium and which had the highest LOD score (critical LOD score 3.0; with a tie the SNP nearest to the Barban SNP) were selected.

### Random negative control SNPs

An initial list of 200 random SNPs was retrieved using random numbers from R *runif*. Locations were found in dbSNP [https://www.ncbi.nlm.nih.gov/SNP/] and those lying on chromosomes 1, 5, 14, X, Y or in the region of the inversion on chromosome 17 (between the two microsatellite markers D17S810 and D17S791), or those without three genotypes, were excluded.

### Script

Included SNPs were analyzed by a custom-made script using the R statistical platform (version 3.4.3 64-bit, https://www.r-project.org^12^; references to all packages are given in Supplemental_file_S1). PLINK was triggered by an R shell command to retrieve data files from the input file. The .ped and .map files created were read into the console and uploaded genotype data was merged with the phenotype data to obtain tables which were analyzed statistically.

### Statistical models

Analyses used zero-inflated negative binomial regression with 0 to 3 children set to zero as non-events. (Note these are non-events as far as the count part of the model is concerned; in the zero's part they would be considered zero "events".) For particular genotypes versus reference genotype:

- model <—pscl::zeroinfl(NCI ~ SNP * Age * sex, data, weights, dist = "negbin").

Weights followed^3^ as the inverse number of subjects in each Age bin, distributed to each subject and scaled to total number of subjects. Overdispersion was checked using the *p*-value for log(theta). If interactions were not significant then interactions were removed. For genotype association:

- car::Anova(model, type = "II", test = "Chisq").

The significance level was set as *p* = 0.05. The inversion SNPs are highly linked and, although analyses of all filtered SNPs were performed, only one SNP is necessary as a representative SNP: this was chosen as rs12373123 (with maximum data). For one model Bonferroni correction therefore included seven SNPs (the inversion representative and the six surrogates for the Barban SNPs) i.e. giving an adjusted significance level of 0.05/7 = 0.007.

### Ethics approval and consent to participate

Database production was approved by the regional ethical committee (Institutional Review Board of the University of Łódź). Genetic data was from anonymous healthy Polish unrelated volunteers who signed written informed consent (see Sobalska-Kwapis et al.^18^).

## Results

The POPULOUS database consisted of 5760 subjects who declared themselves healthy. Based on data from the Polish Central Statistical Office (2012), this represented 0.015% of the Polish population (https://demografia.stat.gov.pl/BazaDemografia/StartIntro.aspx). The ratio of females to males was approximately 50% overall and in each of the 16 districts of Poland. In general the number of subjects enrolled decreased with increasing age (which is one reason why weights were included, as in^3^). The number of subjects with zero children was 1713; one child: 1051; two children: 1641; three children: 747; four children: 316; five or more children: 239, giving an expected mode at two children (see Table 1). The maximum number of children registered per subject was 13 children.

**Table 1:** Numbers of subjects or alleles with each number of children per individual (NCI) for the genotypes of rs12373123, representing the large chromosome 17 inversion.

Genotype	Numbers of subjects or alleles with each NCI:													
	0	1	2	3	4	5	6	7	8	9	10	11	12	13
CC	6	7	19	7	0	1	0	0	0	1	0	0	1	1
CT	65	66	217	107	56	22	14	2	1	1	3	0	0	0
TT	132	218	539	308	132	43	27	6	6	4	2	1	0	0
CT + TT	197	284	756	415	188	65	41	8	7	5	5	1	0	0
CC + CT	71	73	236	114	56	23	14	2	1	2	3	0	1	1
allele_C	77	80	255	121	56	24	14	2	1	3	3	0	2	2
allele_T	329	502	1295	723	320	108	68	14	13	9	7	2	0	0

Genotype	Total number	Mean NCI												
CC	43	2.44												
CT	554	2.35												
TT	1418	2.32												
CT + TT	1972	2.32												
CC + CT	597	2.35												
allele_C	640	2.36												
allele_T	3390	2.32												

Data from males and females combined.

### Inversion SNPs

**Figure 1:**
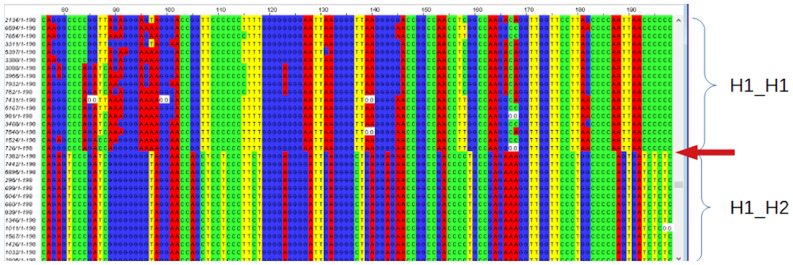
Subjects (each row one subject; anonymized codes in first column) showing non-recombinant groups H1_H1 and H1_H2. Concatenated diploid SNP bases (two adjacent columns for each SNP) were aligned using MAFFT (see methods). Red arrow shows demarcation between subjects with (mostly) homozygous or heterozygous SNP bases. Red: A, adenine; green: C, cytosine; blue: G, guanine and yellow: T, thymine. (The figure shows only a small part of the total alignment).

**Figure 2:**
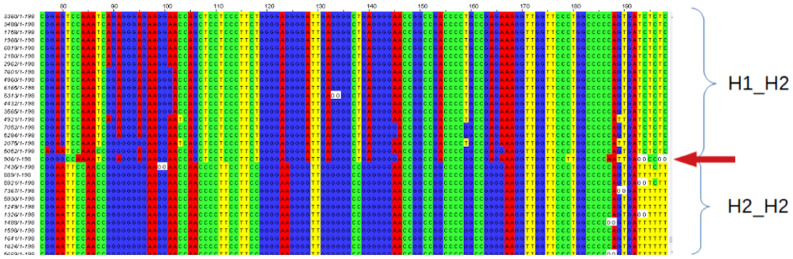
Subjects, as in Fig. 1, aligned with non-recombinant groups and labelled as H1_H2 and H2_H2 groups. (The figure shows only a small part of the total alignment).

SNPs from chromosomal regions at 17q21.31 (see Methods) allowed identification of non-recombinant groups indicating the large inversion; SNPs from Region 1 (177 SNPs) and the enclosed Region 2 (155 SNPs), were selected. Following final exclusions (tertiary exclusions of non-aligned SNPs from Region 2), SNPs which remained were: 118 SNPs for Region 1 and 99 SNPs for Region 2. Only two of these SNPs, rs12373123 and rs12373139, had a coverage of 100% subjects (n = 5470) and these formed identical groupings of homozygous and heterozygote subjects. The first, rs12373123, was chosen as the representative SNP for the H1_H2 inversion. The SNPs rs62054815, rs12185233, rs12185268 and rs12373142 showed exactly the same three groups with less coverage (numbers of subjects not genotyped: 59, 1, 23 and 8, respectively). 16 other SNPs (14 SNPs within the inversion region, 2 SNPs presumed to lie in a flanking region) were automatically arranged into one of the three groups and aligned with the above six SNPs, although some subjects (n = 10) were found to have a contrasting genotype for one or more of these SNPs (assumed to have resulted from mutation subsequent to H1/H2 divergence; borderline subjects were assigned to a group according to maximum similarity). Overall the alignments for these 22 SNPs (the "indicator" SNPs) were very good. The remaining SNPs were manually placed into the alignment without changing the subject order. Figures 1 and 2 show small sections of the delineations (red arrows) between homozygote and heterozygote subjects.

The largest group of subjects (n = 4050, 70.3%), each subject having a homozygous genotype for all of the 22 indicator SNPs, were presumed (see Discussion) to have an [H1][H1] genotype (referred to as the H1_H1 group or "homozygous genotype 1"). The 1561 (27.1%) subjects found to have a heterozygote genotype for all of the indicator SNPs are referred to as the H1_H2 group. The smallest subject group (n = 149, 2.59%) is referred to as the H2_H2 group (or "homozygous genotype 2") and each subject in the H2_H2 group had a complementary genotype to that of subjects in the H1_H1 group for all or most SNPs.

The R coding for zero-inflated negative binomial (with 0 to 3 children set as non-events, Supplemental_file_S1) was designed to analyze many SNPs at once and was run for all the inversion SNPs (data: Supplemental_file_S2; results: Supplemental_file_S3). Statistically significant results (*p* < 0.05) were obtained with 27 (from 69) of these highly-linked SNPs (with male and female data together and with male data alone; female data alone gave only four such results). (13 inversion SNPs did not form a correct model e.g. with only two genotypes after setting non-events, or lack of an interaction model).

However, only one SNP (rs12373123) is necessary to represent the inversion (Table 1) and regression model results for this SNP, adjusted by year of birth and sex, are shown in Table 2. (The initial regression model with (non-significant) interactions is given in Supplemental_file_S8.) For completeness sake, numbers of children from male and female reports separately are given in Supplemental file S8, and regression results are given in each Supplemental results file after those for both sexes combined.

**Table 2:** Results from zero-inflated negative binomial regression of rs12373123 (representing the chromosome 17 inversion) genotypes against numbers of children (NCI), adjusted for year of birth (as Age) and sex; interactions were not significant and were removed.

Pearson residuals:	Min	1Q	Median	3Q	Max
**zeroinfl (formula = NCI ~ SNP + Age + SEX, data, weights, dist = "negbin")**					
	− 0.8199	− 0.4048	− 0.3406	− 0.2595	5.8534

	Estimate	SE	z value	Pr( >|z|)	
**Count model coefficients (negbin with log link):**					
(Intercept)	1.986560	0.259944	7.642	2.13e − 14 ***	
SNP:CT	− 0.508373	0.194147	− 2.618	0.00883 **	
SNP:TT	− 0.557395	0.191392	− 2.912	0.00359 **	
Age	0.001188	0.002895	0.410	0.68149	
Sex	0.028820	0.055795	0.517	0.60548	
Log(theta)	15.476737	35.489470	0.436	0.66277	

	Estimate	SE	z value	Pr( >|z|)	
**Zero-inflation model coefficients (binomial with logit link):**					
(Intercept)	3.906649	0.691704	5.648	1.62e − 08 ***	
SNP:CT	− 0.988095	0.575369	− 1.717	0.0859	
SNP:TT	− 0.700774	0.569460	− 1.231	0.2185	
Age	− 0.027448	0.006922	− 3.965	7.33e − 05 ***	
Sex	0.118935	0.134470	0.884	0.3764	
Theta = 5,265,762.6024; Number of iterations in BFGS optimization: 14					
Log-likelihood: − 1514 on 11 Df					
Analysis of Deviance Table (Type II tests); car::Anova(MODEL, type = "II", test = "Chisq")					

	Df	Chisq	Pr(> Chisq)		
**Response: NCI**					
SNP	2	8.8689	0.01186 *		
Age	1	0.1685	0.68149		
Sex	1	0.2668	0.60548		

Numbers of children 0 to 3 were set as non-events. Significance codes: ***p < 0.001; **p < 0.01; *p < 0.05.

Note that for rs12373123 all of: TTvs.CC, CTvs.CC and genotype gave significant results for association with numbers of children per individual (Table 2). If all models were considered then these did not survive Bonferroni correction; however if only one model was considered then TTvs.CC (*p* = 0.004) did survive Bonferroni correction. The largest numbers of children were found with CC homozygotes (mean 2.4 children per individual, see Table 1), with both CT and TT having less (both means 2.3 NCI; regression count estimates ± standard errors: CT, − 0.508 ± 0.194; TT, − 0.557 ± 0.191; Table 1). A recessive model is indicated by these statistics. Interactions with year of birth and sex were not significant (see Supplemental_file_S8) and were removed from the model. Year of birth and sex effects were not significant but were kept in the model, following^3^. Log(theta) was also not significant, showing that the final model was not overdispersed (it would be quite surprising if it was, as both negative binomial and the zero-inflation are designed to counter this). In the zeros part of the regression model the year of birth was highly significant as expected and no genotype associations were significant.

### Barban SNP results

The three Barban SNPs could not be found in the POPULOUS database and therefore six surrogate SNPs were chosen as detailed in the Methods. One of the highest LOD scores was found for a surrogate SNP almost 35 000 bp away from a Barban SNP, while closer SNPs often gave much lower scores (Table 3). Each surrogate SNP selected was strongly linked to its Barban SNP, with those furthest away presumably having frequently interpositioned double-recombination events rendering lower LOD scores for closer SNPs. (For one Barban SNP, rs13161115, two surrogate SNPs with very high LOD scores were found on the same side of the Barban SNP; see Table 3, and these were analyzed).

**Table 3:** Linkage disequilibrium analyses (using Haploview with the EUR' population) between single nucleotide polymorphisms (SNPs) found by Barban et al.^4^ to be associated with numbers of children (Barban SNPs) and surrogate SNPs which were chosen with the highest logarithm of odds (LOD) scores up to 50 000 base pairs from each Barban SNP.

Barban SNP	Surrogate SNPs	Distance (bp)*	D’	LOD score	r^2^
rs10908474	rs6427268	− 33 035	0.675	20.3	0.286
	rs4434872	20 551	0.953	10.1	0.119
rs13161115	rs2194207	− 16 426	0.972	94.4	0.911
	rs12656808	− 3414	1.00	17.2	0.164
rs2415984	rs10149370	− 23 065	0.957	95.0	0.840
	rs1669786	20 379	1.00	113	0.911

*Estimated distance in base pairs (bp): minus = upstream of Barban SNP; D', Lewontin's D′ statistic; r^2^, Pearson's r-squared statistic.

With negative binomial regression, using identical coding to that used for the inversion SNPs, all SNPs formed a model. No significant associations were found between Barban SNPs and NCI, either for male and female data together or separately (Supplemental_file_S1; data: Supplemental_file_S4; results: Supplemental_file_S5).

### Random negative control SNPs

After exclusions, 109 negative control SNPs were analyzed using zero-inflated negative binomial regression using identical coding as above. Models were not formed with 11 SNPs, leaving 98 SNPs as negative controls. No significant associations were found between these SNPs and NCI for male and female data together or for male data alone. However, for female data, one SNP: rs4485340, did give a positive result for GA vs. AA, *p* = 0.0261 (data: Supplemental_file_S6; results: Supplemental_file_S7).

## Discussion

Although a recessive model is indicated by the data, a dominant descriptive statistic can be created to compare with that given by Stefansson et al.^3^: With reports from both sexes combined, homozygotes/heterozygotes with the H2 allele were estimated to have 0.0796 more children in the Icelandic population^3^; and 2.353 − 2.315 = 0.0380 more children in the Polish population (from the present study). Together with the significant regression result this can be regarded as positive confirmation that the inversion is associated with more children. The recessive model, indicated by the results to be more appropriate, gave 2.442 − 2.324 = 0.118 more children for Polish H2 homozygotes.

The prevalence of H2 haplotypes, as identified by SNP non-recombinant groups, was estimated as 15.9% in the Polish population (from Table 1: number of C alleles / total number of alleles), which compares with frequencies of H2 microsatellite haplotypes found by Stefansson et al.^3^: 1% (n = 66) in subjects of Asian descent, 6% (n = 177) with relatively recent African descent and 21% (n = 241) with European descent.

As mentioned in the Introduction, similarities or differences between phenotypes should be considered. The phenotype studied in the present article is the number of children per individual (NCI). Several researchers have defined the "number of children ever born" (NEB; meaning how many conceptions the woman had including still-births and fetal deaths) (e.g. ^3^.). However, for various reasons including cultural sensitivities, it is not always possible to get correct answers to questions which concern still-births, especially if a man is asked the question. In 2019 the numbers of stillbirths in Poland was estimated at ~ 0.2%^19^ and this is unlikely to affect association outcomes except via interactions with age (which analyzes identically to year of birth; this is of importance with decreases in infant mortality over time).

Additionally, as well as the obvious uncertainties in data derived from male responses, there is also uncertainty inherent with parental age: young people might have more children after the survey. Fertility rate falls with age^20^ and data was therefore used from minimum ages following^4^. Note that even the age at which women remain fertile might change with year of birth as the fecundity period of women appears to be lengthening^21^.

A second feature to be considered is the regression model. In the present study Poisson-related regressions were utilized, following Ibeji et al.^22^. These have numerous advantages over linear regression, not least that in linear regression a fundamental assumption often only loosely followed is that the relationships should be somewhat linear (!) which is not the case if there is a peak at two or more children. (The female H2 data in Stefansson et al.^3^ mentioned in the Introduction was configured to have near-linearity, but other genotypes almost certainly had peaks at two children, as with the male H2 data.) Poisson-related distributions are designed for count data and the negative binomial is the least subject to over-dispersion. (Zero-inflated models also provide a further counter to over-dispersion).

Perhaps surprisingly, there is no evidence whatsoever that sex has an effect on the phenomenon considered, for two reasons: (1) in two studies now the main sex effects were not significant, and (2) casual observation of results from separated sexes (disregarding non-significant SNP main effects, and small amounts of data for separated sexes in the present study) might lead to a guess that sex has exactly opposite effects in the two populations—which is a highly unlikely scenario. The lack of effect of sex is another reason for supposing that any causal relationship is more likely to be to do with human reproductive behavior than, for example, effects on male fertility.

The association does not give any direct evidence for a causal effect, as the inversion could well be correlated with other aspects of the genome. However, it is already known that the inversion, and/or genes within it, have various associations with neurological effects. For example, the H1D and H2D versions studied by Steinberg et al.^11^ and highly prevalent in Europeans (note it was not possible to distinguish these from the less common H1' and H2' versions in the present study) have been associated with protection against various neurological disorders such as Parkinson’s disease, Alzheimer’s disease, and progressive supranuclear palsy^23,24^. The H2D haplotype has also been associated with a microdeletion syndrome causing developmental delay and epilepsy, among other clinical characteristics, in ~ 1/16,000 Europeans^25^.

Additionally, the inversion contains at least five genes; *KANSL1*, *NSF*, *MAPT*, *CRHR1* and *SPPL2C*, all of which have been associated with neurological conditions. This functional clustering is either coincidence, might reflect structural/functional organization of the human genome, or perhaps some functional associations have been misinterpreted due to strong linkage with other genes (?). These genes are described below.

1. A similar disease phenotype to microdeletion syndrome is found with haplotype insufficiency in the *KANSL1* gene, thought to be involved with neuronal function^26^. Recurrent partial duplications of the *KANSL1* gene have occurred on both H1 and H2 haplotypes and have risen to high frequency in European populations. Steinberg et al.^11^ confirmed three haploid copy number states for the *KANSL1* locus.
2. The *NSF* locus also has three haploid copy number states and with a copy number of two, the *NSF* gene (which codes for Vesicle-fusing ATPase) has one copy which lies outside of the inversion and one (with the first 13 exons only) inside—which is therefore inverted in H2. Mutations in the *NSF* gene cause infantile epileptic encephalopathy^27^.
3. The *MAPT* (Microtubule-associated protein tau) gene was found to have two highly divergent H1 and H2 variants and strong linkage disequilibrium, and this gene has been implicated in, for example, Parkinson’s disease, Alzheimer’s disease, and progressive supranuclear palsy^23,24,28–30^.
4. The *CRHR1* gene is unusual in that very similar segments exist on H1 and H2 which are thought to be too large to be due to gene conversion; Steinberg et al.^11^ concluded that a double recombination had taken place, probably before the dispersal of modern humans out of Africa. The *CRHR1* gene has been implicated in brain function and anxiety^31^.
5. Lastly, the *SPPL2C* gene (which encodes Signal peptide peptidase-like 2C = Intramembrane protease 5), has been associated with Parkinson’s disease^32^.

It is therefore quite possible that combinations of versions of the above genes have neurological effects on human reproductive behavior, although this would need to be established. Establishing causal relationships between behavior and genetics is, at present, thought to be exceptionally difficult (see^33^). Less likely, although possibly in combination with behavioral effects, there might also be some relationship with male fertility, although the genes mentioned above do not seem to suggest this.

There are numerous systematic differences between the result presented here and that from Stefansson et al.^3^, any one of which, or any combination, might theoretically have led to misleading results or a false positive in either study, but are now, following replication, thought not to have done so. Non-biological differences include: (1) the definition of phenotype; (2) the source of data, including whether the subjects were related or unrelated; (3) the number of subjects (which might contribute to random effects); (3) the age ranges chosen for analysis; and (4) the regression model. Therefore, the fact that effects have been shown in both studies, despite these (and many other possible) differences, leads to the conclusion that there is indeed some sort of effect of the inversion on numbers of children. Further replication is also warranted, especially to confirm the more advanced regression model used in the present study.

Limitations of the present study: in further replication it would be advisable to (1) switch to or confirm data using genealogy databases rather than questionnaires; (2) include phenotype data for obesity, excessive exercise, smoking, alcohol consumption and sexually transmitted diseases to be used as adjustors; and (3) increase the sample size to nearer that in the Stefansson et al.^3^ study. Perhaps surprisingly, even though an initial base of almost 6000 subjects were assessed, age/genotype divisions reduced the numbers of H2_H2 homozygotes, and a future sample size of 25 000 could be recommended. The prevalence of the H2 haplotype was (relatively) quite high in the Polish population, and as this might also be true of related Slavic populations, a study of Eastern European populations might possibly give similar results. It is also worth considering how much data would need to be collected in order to confirm the previous studies without concerns relating to changes in societal pressures (and other changes) over time. To parallel the Stefansson et al.^3^ study but with only male data selected from a one-year age group, e.g. men aged 55, would require a population study with data collected similarly to the present study of around five million people. Interestingly, GWASs with over one million subjects are starting to be processed^34^, so with time such data may become available for analysis.

Using sequencing it would be possible to confirm the H2 genotype, but although this was not confirmed in the present study there are no countries in the world which have an H2_H2 predominance and H2 is consistently the minor allele. However, sequencing would also be useful to distinguish genotype subtypes and especially to measure the prevalence of H2D and H2'.

No conclusions can be made concerning the Barban SNPs, especially as surrogate SNPs were analyzed. Future bead-chip arrays might include the exact SNPs, or next-generation sequencing would also improve the chances of showing association, if one indeed exists, in the population.

In conclusion, notwithstanding caveats regarding different methodologies between the present study and that of Stefansson et al.^3^ in the Icelandic population, an association has been replicated between the H2 haplotype of the large inversion on chromosome 17 at 17q21.31 and increased numbers of children in the Polish population, providing some evidence that this association applies at least to European populations. Although five genes in the inversion are likely associated with neurological effects, relationships between these and human reproductive behavior (or even, perhaps, male fertility) have yet to be established.

## Supplementary Information

Supplementary Information 1.

Supplementary Information 2.

Supplementary Information 3.

Supplementary Information 4.

Supplementary Information 5.

Supplementary Information 6.

Supplementary Information 7.

Supplementary Information 8.

## Data Availability

All data is found in Supplemental materials and is also found at https://github.com/Abiologist/InversionSNPs.git.
