# Supplementary material for: Association studies between chromosomal regions 1q21.3, 5q21.3, 14q21.2 and 17q21.31 and numbers of children in Poland
Source: Sci Rep. 2022 Nov 7;12:18923. doi: 10.1038/s41598-022-21638-x (PMC9640534; doi:10.1038/s41598-022-21638-x)
Supplement: Supplementary file 8 — Supplementary Information 8. [file 41598_2022_21638_MOESM8_ESM.docx]

Supplemental file S8 for article:

Title: "Association studies between chromosomal regions 1q21.3, 5q21.3, 14q21.2 and 17q21.31 and numbers of children in Poland."

A, B. Numbers of children per individual (NCI) for the genotypes (CC, CT and TT) of representative inversion SNP rs12373123 ((which represents the inversion on chromosome 17), with recessive and dominant divisions. A. Reports from females only. B. Reports from males only.

C. Zero-inflated negative binomial regression with (non-significant) interactions, for male and female data together.

**A. Reports from females only.**

|  | **Numbers of subjects or alleles with each NCI** | | | | | | | | | | | | | |
| --- | --- | --- | --- | --- | --- | --- | --- | --- | --- | --- | --- | --- | --- | --- |
| **NCI** | **0** | **1** | **2** | **3** | **4** | **5** | **6** | **7** | **8** | **9** | **10** | **11** | **12** | **13** |
| **CC** | 6 | 5 | 12 | 4 | 0 | 1 | 0 | 0 | 0 | 1 | 0 | 0 | 0 | 0 |
| **CT** | 43 | 32 | 147 | 62 | 28 | 10 | 9 | 1 | 0 | 1 | 1 | 0 | 0 | 0 |
| **TT** | 97 | 138 | 317 | 176 | 87 | 32 | 13 | 3 | 2 | 2 | 1 | 0 | 0 | 0 |
| **CT+TT** | 49 | 37 | 159 | 66 | 28 | 11 | 9 | 1 | 0 | 2 | 1 | 0 | 0 | 0 |
| **CC+CT** | 140 | 170 | 464 | 238 | 115 | 42 | 22 | 4 | 2 | 3 | 2 | 0 | 0 | 0 |
| **allele_C** | 55 | 42 | 171 | 70 | 28 | 12 | 9 | 1 | 0 | 3 | 1 | 0 | 0 | 0 |
| **allele_T** | 237 | 308 | 781 | 414 | 202 | 74 | 35 | 7 | 4 | 5 | 3 | 0 | 0 | 0 |

|  | **0 to 13** | **Mean NCI** |
| --- | --- | --- |
| **CC** | 29 | 1.90 |
| **CT** | 334 | 2.26 |
| **TT** | 868 | 2.25 |
| **CT+TT** | 363 | 2.25 |
| **CC+CT** | 1202 | 2.23 |
| **allele_C** | 392 | 2.20 |
| **allele_T** | 2070 | 2.25 |

**B. Reports from males only.**

|  | **Numbers of subjects or alleles with each NCI** | | | | | | | | | | | | | |
| --- | --- | --- | --- | --- | --- | --- | --- | --- | --- | --- | --- | --- | --- | --- |
| **NCI** | **0** | **1** | **2** | **3** | **4** | **5** | **6** | **7** | **8** | **9** | **10** | **11** | **12** | **13** |
| **CC** | 0 | 2 | 7 | 3 | 0 | 0 | 0 | 0 | 0 | 0 | 0 | 0 | 1 | 1 |
| **CT** | 22 | 34 | 70 | 45 | 28 | 12 | 5 | 1 | 1 | 0 | 2 | 0 | 0 | 0 |
| **TT** | 35 | 80 | 222 | 132 | 45 | 11 | 14 | 3 | 4 | 2 | 1 | 1 | 0 | 0 |
| **CT+TT** | 57 | 114 | 292 | 177 | 73 | 23 | 19 | 4 | 5 | 2 | 3 | 1 | 0 | 0 |
| **CC+CT** | 22 | 36 | 77 | 48 | 28 | 12 | 5 | 1 | 1 | 0 | 2 | 0 | 1 | 1 |
| **allele_C** | 22 | 38 | 84 | 51 | 28 | 12 | 5 | 1 | 1 | 0 | 2 | 0 | 2 | 2 |
| **allele_T** | 92 | 194 | 514 | 309 | 118 | 34 | 33 | 7 | 9 | 4 | 4 | 2 | 0 | 0 |

|  | **0 to 13** | **Mean NCI** |
| --- | --- | --- |
| **CC** | 14 | 3.57 |
| **CT** | 220 | 2.48 |
| **TT** | 550 | 2.42 |
| **CT+TT** | 770 | 2.44 |
| **CC+CT** | 234 | 2.55 |
| **allele_C** | 248 | 2.61 |
| **allele_T** | 1320 | 2.43 |

C. For males and females together, zero-inflated negative binomial regression with interactions (not significant).

$rs12373123

Call:

zeroinfl(formula = NCI ~ p[, 5] * Age * SEX, data = p, weights = myweights,

dist = "negbin")

Pearson residuals:

Min 1Q Median 3Q Max

-1.0181 -0.3923 -0.3372 -0.2683 5.5564

Count model coefficients (negbin with log link):

Estimate Std. Error z value Pr(>|z|)

(Intercept) 11.47871 10.32723 1.111 0.266

p[, 5]CT -11.21003 10.37177 -1.081 0.280

p[, 5]TT -10.67173 10.35500 -1.031 0.303

Age -0.15343 0.16302 -0.941 0.347

SEX -4.93599 7.74792 -0.637 0.524

p[, 5]CT:Age 0.17662 0.16369 1.079 0.281

p[, 5]TT:Age 0.16308 0.16345 0.998 0.318

p[, 5]CT:SEX 5.58233 7.78284 0.717 0.473

p[, 5]TT:SEX 5.64189 7.77353 0.726 0.468

Age:SEX 0.08457 0.12767 0.662 0.508

p[, 5]CT:Age:SEX -0.09612 0.12814 -0.750 0.453

p[, 5]TT:Age:SEX -0.09414 0.12803 -0.735 0.462

Log(theta) 16.10024 20.39988 0.789 0.430

Zero-inflation model coefficients (binomial with logit link):

Estimate Std. Error z value Pr(>|z|)

(Intercept) 34.1875 22.0165 1.553 0.120

p[, 5]CT -32.5590 22.1597 -1.469 0.142

p[, 5]TT -29.8339 22.0804 -1.351 0.177

Age -0.5103 0.3608 -1.414 0.157

SEX -24.4181 19.7673 -1.235 0.217

p[, 5]CT:Age 0.5111 0.3630 1.408 0.159

p[, 5]TT:Age 0.4617 0.3618 1.276 0.202

p[, 5]CT:SEX 26.4900 19.8645 1.334 0.182

p[, 5]TT:SEX 23.1295 19.8149 1.167 0.243

Age:SEX 0.3945 0.3317 1.189 0.234

p[, 5]CT:Age:SEX -0.4304 0.3331 -1.292 0.196

p[, 5]TT:Age:SEX -0.3704 0.3324 -1.114 0.265

Theta = 9822987.3604

Number of iterations in BFGS optimization: 52

Log-likelihood: -1501 on 25 Df
